# Chemosensory Changes from Cancer Treatment and Their Effects on Patients’ Food Behavior: A Scoping Review

**DOI:** 10.3390/nu11102285

**Published:** 2019-09-24

**Authors:** Alissa A. Nolden, Liang-Dar Hwang, Anna Boltong, Danielle R. Reed

**Affiliations:** 1 Food Science Department, College of Natural Sciences, University of Massachusetts, Amherst, MA 01003, USA; 2 Monell Chemical Senses Center, 3500 Market Street, Philadelphia, PA 19104, USA; reed@monell.org; 3 The University of Queensland Diamantina Institute, The University of Queensland, Woolloongabba, Queensland 4102, Australia; danielldhwang@gmail.com; 4 Victorian Comprehensive Cancer Centre, Melbourne 3000, Australia; anna.boltong@unimelb.edu.au; 5 Melbourne School of Population and Global Health, The University of Melbourne, Parkville 3053, Australia

**Keywords:** chemotherapy, chemosensory loss, dysgeusia, chemosensory dysfunction, chemosensory perception, flavor, taste, smell, appetite, oncology, dietician

## Abstract

Individuals undergoing treatment for cancer can experience changes in taste or smell that are often assumed to affect constructs related to food behavior, although this relationship is rarely measured directly. To ascertain the extent to which measured changes in taste and smell during and after cancer treatment affect food behavior, we conducted a scoping review and completed a comparative analysis for studies that met our criteria, which were: they directly measured cancer patients’ (a) psychophysical response to taste and/or olfactory stimuli, and (b) food behavior (including food enjoyment, food preference, dietary intake) in people affected by cancer. Eleven studies met these criteria and were included in the review. All 11 studies evaluated taste and five also measured smell. A comparative analysis exploring taste and food behavior shows that a reduced sweet taste function (decreased sensitivity) was associated with a reduced intake of a variety of different macro and micro nutrients, reduced appetite, and overall lower energy intake. One out of six studies that measured smell and food measured observed changes in olfactory function following cancer treatment. There were no significant relationships reported between olfactory measures and food behavior. Taste changes that arise from cancer treatment appear to have a direct effect on food behavior, although there is a need for more research using standardized measures and larger sample sizes. A better understanding of taste alterations and their implications for dietary intake and food enjoyment will support optimal nutritional health by identifying strategies to help patients eat well during and after cancer treatment.

## 1. Introduction

According to the National Cancer Institute, in the United States, one in three adults are diagnosed with cancer (based on 2013–2015 data) [1]. The decline in the cancer death rate (26% reduction between 1991 and 2015) [1], is resulting in a larger number of individuals surviving cancer, with the number of cancer survivors expected to rise to 20.3 million by 2026 [2].

Many people receiving cancer treatment complain of changes to their taste and smell functions, presenting as various manifestations, though routinely described as a loss or alteration of perception [3]. Prevalence of self-reported taste problems among cancer patients ranges from 12% to 84% [4,5,6]. Taste and smell play an important role in food behavior, so changes in taste or smell perception may negatively influence eating behavior [7,8,9]. “Food behavior” is an inclusive term to capture any type of food-related behavior that might have real-world significance for patients’ overall nutritional health [10], such as the desire to eat, food preferences, and food intake, in ways that lead to weight change and poor quality of life [11,12,13,14]. Several studies have investigated cancer-related changes in chemosensation (i.e., taste and smell) or food-related behavior; however, it is rare for a study to use both measures.

There is a wide variability in the prevalence rates for reported taste function (12% to 84% [4,5,6]). There are both clinical and demographic patient characteristics that could have an impact on taste function. The various treatment regimens and cancer types studied, the different methods used to evaluate taste [3,15,16], how “taste” was defined in these studies, and the end points examined, all effect the study results. For example, some authors describe “taste” as a study end point but do not assess taste per se. Other patient characteristics, such as smoking history, alcohol abuse, and genetics, have all known to play a role in individual variability in taste perception.

This review critically evaluates research published from 1982 to 2018 that psychophysically measured taste and smell function and assessed some aspect of food behavior (e.g., appetite, dietary intake, and preference). We reviewed studies that used objective measures of taste and smell function (i.e., using psychophysical methods), as opposed to self-reported changes in taste or smell. The rationale for this selection strategy was based on the observation that patients often do not distinguish between taste and smell or confuse sensory constructs with hedonic constructs, such as appetite or food enjoyment, so self-reported changes are often unrelated to objectively measured taste changes [17,18]. For example, a food may taste the same as it always did but that taste is no longer enjoyed.

## 2. Methods

### 2.1. Study Selection

Our focus on taste and food behavior in people with cancer posed several challenges in systematically reviewing the literature because no standard search terms returned appropriate studies. We therefore took a two-pronged approach: First, we started with the most current published papers and traced citations back to 1961, the earliest paper reporting on taste psychophysics of cancer patients. This paper from 1961 served as the starting point of the period covered by the review. Second, we included the broad terms “taste” and “smell” (i.e., taste, smell, olfaction, chemosensory), “objective evaluation” (i.e., threshold, detection threshold, recognition threshold, sensitivity, intensity, identification), “food behavior” (i.e., dietary food intake, food preference, food behavior, appetite, food liking), “human,” and “cancer” or “chemotherapy” and read the abstracts to identify relevant papers. Papers were removed that did not use methods that objectively measured taste and/or smell function or did not include measures of food behavior. Papers were not excluded based on study design (e.g., cohort or longitudinal), cancer diagnosis, treatment type, or time since cancer diagnosis or treatment. Papers that met the above criteria were removed if articles were not written in English, included children (<18 years old), or were not original research publications. Database searches were carried out in PubMed, Web of Science, Google Scholar, and from the University of Pennsylvania and University of Massachusetts Libraries. Two authors reviewed the abstracts of the unique original research articles from the resulting literature searches (*n* = 44), and additional articles were identified through reference searches and searches of similar articles, with 11 articles fulfilling the criteria.

### 2.2. Data Extraction

Data from selected articles were manually extracted, including sample size, experimental design, methods of sensory measurement, food behavior metrics, and summary statistical statistics.

### 2.3. Statistical Approach for Comparative Analysis

Three of the eleven articles were included in the comparative analysis due to eight of the eleven articles having insufficient data available for extraction. Means and standard deviations of the comparison groups, test statistics, and other metrics for the comparative analysis were obtained where possible. We calculated the effect sizes with the *d* statistic using previous methods [19].

## 3. Results

### 3.1. Study Characteristics and Design

The characteristics of the 11 studies included in the review and comparative analysis are summarized in Table 1. The studies included had a combined total of 578 participants, comprising 380 people with cancer and 198 controls. Five studies enrolled patients with more than one cancer type and six enrolled patients with a single cancer type. Cancer diagnoses varied across studies, including breast, esophagogastric, lung, ovarian, prostate, colon, multiple myeloma, lymphoma, and testicular cancer, with one study not reporting a cancer type. 

### 3.2. Methods of Taste and Smell Assessment

A variety of objective measures were used to evaluate taste and smell perception. For taste, procedures included detection threshold (DT) and recognition threshold (RT). DT is the lowest concentration that a taste stimulus can be detected compared to the background (i.e., different than water), with RT referring to the lowest concentration a specific taste can be recognized (e.g., bitter). Other taste methods included taste identification tasks evaluating the suprathreshold stimuli (e.g., International Organization for Standardization (ISO) 3972:2011) intensity rating of suprathreshold stimuli (concentration above DT), and ranking tasks. Of note, the detection threshold occurs at a lower concentration than the recognition threshold. Most studies reported stimuli being presented at room temperature with participants having time between stimuli where water was available for rinsing. The studies also differed in the way investigators presented smell and taste stimuli to subjects. For taste, stimuli were presented to either the whole mouth (whole-mouth rinses, sprays) or only parts of the mouth (filter paper disks, taste strips). For smell, most of the studies used Sniffin’ Sticks (a set of pen-like devices that dispense an odor) to make three measures of olfaction function: identification, sensitivity (DT), and discrimination [29]. Table 2 summarizes the methods used to evaluate taste and smell. For a general review of methods evaluating taste and smell function in oncology populations, see References [30,31,32,33]; for more methodological detail, see References [34,35]; and for clinical applications for dietitians, see Boltong and Campbell [36].

A variety of different methods were used to assess food behavior across studies. Eight studies included measures of dietary intake, assessed using either a dietary recall (*n* = 2), a food frequency questionnaire (*n* = 5), or a 3-day weighed food technique (*n* = 1). Participants answered questions regarding symptoms related to appetite (*n* = 5); however, only two studies analyzed the relationship between chemosensory measures and appetite. Four studies measured food preferences using responses to food pictures with different macronutrient content.

### 3.3. Impact of Taste Changes on Food Intake and Preferences

When comparing the experimental results between taste and food behavior for each type of study design, i.e., cross-sectional and prospective longitudinal, the results were mixed. Of the cross-sectional cohort studies (including one combined cross-sectional and longitudinal study), several reported a significant relationship between taste function and food behaviors. Investigators reported that cancer patients with a loss of appetite were more likely to prefer lower sweetness levels and a reduced intake of sweet foods compared with patients without a reduced appetite [21]. In another study, changes in bitter DT correlated with the avoidance of meat, chocolate, and tea [21]. In a third study, changes in bitter DT correlated with the avoidance of meat, chocolate, and tea; however, the direction (i.e., increased or decreased bitter sensitivity) was not reported [23]. In a fourth study, patients with a higher sucrose DT consumed less protein, carbohydrate, zinc, and fewer calories. In the same study, patients with a higher urea RT reported consuming fewer calories and less protein, carbohydrate, and fat. Sucrose and urea thresholds were also associated with energy intake: a greater percentage of patients who did not meet energy requirements had thresholds above the median for sucrose (DT and RT) and urea (RT) [24], meaning they found it harder to recognize sweet and bitter tastes. The one cross-sectional cohort study did not report a significant relationship between taste function and food behavior in those who had cancer but were in remission and no longer undergoing treatment (cancer survivors; 1–7 years post-treatment), even though changes in taste function were apparent in this group [18].

Among the seven prospective longitudinal studies (including one cross-sectional longitudinal study using a combined approach), three reported a significant relationship between taste and food behavior. In one of these three studies, the reduced ability to identify taste solutions was associated with decreased energy intake during the beginning of the third chemotherapy treatment cycle [25]. In a second study, patients who became more sensitive to sucrose between the start of chemotherapy and after two cycles of chemotherapy consumed fewer grams of protein, animal protein, fat, and iron compared to those who became less sensitive to sucrose [26]. The third study also found a relationship between sweet sensitivity and food behavior, reporting that decreased sucrose sensitivity was associated with reduced appetite prior to treatment [20].

Of the four prospective longitudinal studies that found no taste effects on food behavior, one showed that changes in sensitivity (as measured by the ability to detect electrical stimulation of the tongue) was not associated with changes in chemotherapy patients’ protein or energy intake [22]. In two studies, recognition thresholds did not correlate with food preferences [28] or with the liking of macronutrient categories [17]. The fourth study measured recognition thresholds and collected data on food frequency intake and food preferences but did not report on associations between objective taste and food measures [27].

We computed effect sizes from the studies listed in Table 1. Where possible, we computed these values and report them as *d* (standardized mean difference; Table 3) to assess the magnitude of taste changes, allowing for the comparison of findings across studies. However, 5 of the 11 studies reported no significant effects, and 3 of the 6 studies with significant effects reported too little information to allow us to compute effect sizes. Therefore, only three of the studies were included in the effect size calculations, and thus conclusions should be drawn cautiously. However, the effect sizes revealed that sweet taste has the most empirical evidence for involvement with food behavior, with greater evidence supporting this link. There was some but limited evidence that bitter taste was involved with measures of nutrient intake. Almost all studies measured taste changes for four taste qualities, sweet (*n* = 10), sour (*n* = 8), bitter (*n* = 10), and salty (*n* = 8), with fewer reporting responses to umami (*n* = 3); but positive results and reportable effect sizes were mostly for sweet taste. 

### 3.4. Impact of Smell Changes on Food Intake and Preferences

Five studies measured smell function; three of the five studies reported no significant change among subjects affected by cancer [18,22,28]. One longitudinal study reported significant differences (poorer smell function) for overall olfactory scores and for threshold olfactory scores (but not discrimination or identification tasks) during chemotherapy compared with controls [17]. A prospective cohort study of testicular cancer patients reported lower smell thresholds (greater smell sensitivity) compared with controls at baseline, with no differences at any other time point (through 1-year post-treatment) [27]. Another longitudinal study reported just under 50% (13 of 27) of patients had changed smell detection thresholds (compared to baseline): almost equal numbers of participants experienced increased sensitivity (*n* = 6) and decreased sensitivity (*n* = 7) [22]; no other analysis was reported. No comparative analysis was performed as none of these studies report an instance where smell alterations were significantly related to any measures of food behaviors.

## 4. Discussion

### 4.1. Taste but Not Smell May Influence Food Intake and Enjoyment

The studies reviewed here suggest that cancer patients may suffer from reduced food intake and appetite, which are associated with alterations in taste perception. Specifically, reduced taste perception may result in a decreased intake of fat and protein, leading to an overall decrease in energy consumption (Table 3). These data suggest that patients suffering from reduced taste sensitivity (i.e., higher DT) experience greater incidences of food avoidance. Among the 11 research articles reviewed, six reported no significant relationship between measures of taste (or smell) and food-related behaviors; none of which supported a relationship between smell function and food behavior.

### 4.2. The Special Role of Sweet Taste Changes and Food Behavior in Cancer Patients

We performed a comparative analysis and learned that changes in sweet and, to a lesser extent, bitter perception were more common than changes to salt or sour perception in cancer patients, and that these changes in sweet taste perception were often tied to differences in food behaviors (Table 3). The effect sizes, reported in Table 3, allow for the comparison across the studies reporting a significant relationship between taste and food behavior. The greatest effect size reported was between sweet sensitivity and zinc intake, with individuals having a greater sensitivity for sweetness (i.e., low DT) associated with consuming more zinc, compared to those with a lower sensitivity for sweetness (i.e., high DT). For bitterness, the highest effect size was observed for the daily consumption of calories, with individuals with a higher DT (lower sensitivity) consuming on average 631 fewer calories compared to individuals with a lower DT (greater sensitivity). Reduced sweet taste function (decreased sensitivity) was associated with a reduced intake of a variety of different macro and micro nutrients, reduced appetite, and overall lower energy intake. This suggests that reduced taste function, specifically for sweetness, reduces patient’s food intake.

There are a few mechanisms in which cancer treatment may affect taste and ultimately food behavior. It is plausible that sweet taste receptors or cells are more vulnerable to cancer treatment or that neural response to sweet ligands is altered as a result of cancer treatment. Another consideration is that taste receptors are also expressed in the gut [37] and may be affected in a similar mechanism as oral taste cells during chemotherapy treatment. Therefore, changes in food behavior may be a result of gastrointestinal symptoms that co-occur with taste alterations [38,39]. While caution is warranted in drawing conclusions from this comparative analysis because of the small number of studies, it is clear that these perceptual changes warrant more study.

Taste and smell loss related to cancer treatment is thought to arise as a result of the cancer treatment [40]; yet individuals may experience taste and smell changes prior to treatment as a result of cancer [32]. Each type of cancer treatment may affect taste and smell through different mechanisms [16]. For example, taste loss during chemotherapy may be due to the disruption of taste cell renewal [41,42], whereas surgery in the oral cavity could damage chemosensory nerves [43].

### 4.3. Contrasting Study Designs, Patient Populations, and Methodologies

Cancer treatment is continually making advancements, so as technology and precision medicine advances, toxicities may differ. For example, in the treatment of head and neck cancers, proton radiation has a smaller radiation field, and thus may better preserve gustatory tissue when compared to conventional photon radiation treatment [44]. While it is likely that treatment characteristics and cancer diagnosis may differentially effect taste perception, conclusions cannot be drawn here about the effects of specific treatments on taste and food behavior because of both the diversity of treatments and the limited treatment information provided. Other aspects that could explain the conflicting results are the mix of study designs (see Table 1) and patient population. Diverse patient populations were used across studies, with six studies studied a single cancer type, three studies involved patients from two or more cancer type sub groups, and two studies did not disclose cancer diagnosis. Clinical and demographic characteristics, such as gender, age, smoking history, upper airway infections, and drug use, among other factors, are known to impact chemosensory function; however, these data were reported infrequently and could have had a significant impact on the study outcomes and their interpretations. Moreover, occurrence and severity of treatment-related symptoms, such as dysphagia, dry mouth, and nausea, among others, can negatively impact food behavior [36]. More work is needed to fully understand the implications of co-occurrence of these symptoms on food behavior and overall nutrition. Data and analyses were reported for the study population, regardless of the number of treatment types or cancer diagnoses, making it difficult to conduct a meaningful analysis to examine these clinical characteristics and their association with or influence on taste alterations and food behavior.

As summarized in Table 2, different methods were used to evaluate chemosensory function, which included variations in the taste stimulus delivery method. The authors of several studies did not report key information about the testing procedure, such as the concentration of the taste stimuli, and these methodological details may have contributed to the range of study results. These different methods allow stimuli to contact different areas of the oral cavity and tongue (e.g., sensitivity on the posterior versus anterior tongue), and the delivery method may influence taste perception [45,46]. Because different areas are innervated by different cranial nerves, they may be differentially impacted by treatment [43,47]. Food-related measures also varied across studies. For those evaluating intake, all studies used either food-frequency questionnaires or dietary recalls (24-h or 3-day recalls), with the exception of one weighed food study [23]. Evaluation of food preferences in the four studies used pictures of foods that were representative of portion sizes and different macronutrients [17,18,27,28], developed as a measure of food preference based on taste and macronutrient categories [48]. Studies using this method had mixed results for changes in food preferences during treatment [17,28,49], and were not associated with objective taste and smell measures [17,18,27,49]. These methods are not without limitations and may not accurately represent daily intake. Furthermore, food intake was not always normalized for body weight or accounted for recent weight history. This can be challenging as recommended caloric intake during cancer treatment may not be reflective their current body weight, with clinicians modifying patients’ diet to promote weight loss or maintain weight. Overall, it is recommended that more objective measures be used to quantify food intake [50], notwithstanding the practical barriers and participant burden inherent in doing so.

### 4.4. Individual Differences and Genetic Background

Some of the different findings among studies might be due to variation arising from individual differences in taste perception. A well-studied phenomenon is the wide variation across individuals in response to the bitterness of propylthiouracil (PTU or PROP) and phenylthiocarbamide (PTC) [51,52,53]. Some of the variation in response to these bitter compounds and others is linked to genetic differences in bitter taste receptor genes (*TAS2Rs*) [54,55,56,57,58,59,60]. Individual differences in perception are associated with differences in the liking and intake of foods [60,61,62,63,64]. This points to the importance of individual differences in perception that may result from genetic differences [59,65] (see Hayes et al. [66]) or dietary exposure [67,68,69,70], and is why longitudinal studies are preferred, where the patients act as their own control. Indeed, there appears to be a link between the genetics of a bitter taste receptor (*TAS2R38*) and the risk of developing cancer; however, the direction of this relationship is unclear [71,72,73]; although the direct pathophysiology is not known, it is likely driven by factors other than differences in vegetable and fruit intake [74]. Taken together, greater attention to differences across individuals arising from taste genotypes is warranted regarding taste studies.

### 4.5. Translation to Real-World Food Interactions and Clinical Applications

The goal of this review was to determine whether alterations in chemosensory function are associated with changes in food behavior, including consumption, preference, and enjoyment, in cancer patients. However, we must consider how changes in taste thresholds (DT or RT) translate to how we experience food. Threshold measures are variable and subject to false positives [75], and do not correlate with suprathreshold ratings or hedonic response [33,76,77]. However, the intensity and hedonic response of suprathreshold stimuli has been associated with the intake of a variety of foods and beverages [67,68,78,79]. Of the eleven studies included in this review, three presented taste stimuli at suprathreshold levels [17,21,25], of these studies, two reported a significant relationship with reduced energy intake [21,25], and another reported no relationship with food preference [17]. Selection of the psychophysical sensory methods is important and the translation of these measures to food behavior and nutritional status must be taken into consideration. More studies are needed to evaluate taste function above the detection threshold and how changes in taste perception mediate dietary intake and food behavior.

Patients should receive information on taste changes prior to treatment; this requires the proper training of clinicians such that they are equipped with validated evaluation measures. However, there is no such rapid detection or diagnosis tool that can be administered in a clinical setting. Currently, there are no clinical guidelines on the assessment or strategies for managing or improving taste problems. Future work will help to identify whether patient or clinical characteristics are associated with an increased risk for developing taste alterations, with more work needed to explore the long-term effects of cancer treatment, whether taste fully recovers, or whether there are long-lasting and persistent alterations. Another area that needs attention is the taxonomies used to describe alterations in taste function [15], with disparities being present in the use of the classifications across disciplines. Overall, more work is needed make diagnostic tools available for clinicians and information available for patients, along with identifying strategies for the treatment of taste disorders.

## 5. Conclusions

Taste and smell function related to food behavior in people affected by cancer is understudied and the mix of positive and negative results may be due to the mix of measurement methods and experimental designs [3,15,32,80]. However, we can say that among the studies with statistically significant associations between chemosensory function and food behavior (5 out of the 11 reviewed here), reduced taste function, particularly for sweet stimuli, was associated with a reduced appetite, avoidance of certain foods (e.g., meat), and reduced consumption (e.g., overall calories and protein). Additional studies that use a consistent methodology will better elucidate the duration and extent to which taste and smell alterations impact patient outcome.

The results of laboratory studies investigating taste and food behavior may have practical implications. Clinicians lack standardized tools with a high clinical utility to routinely assess and treat taste and smell disorders in oncology patients. A clearer understanding of the effect of taste and smell alterations on elements of food behavior may support the design and testing of clinical strategies and interventional approaches aimed at mitigating the deleterious effects of chemosensory dysfunction as a result of cancer treatment.

## Figures and Tables

**Table 1:** Summary of studies using direct measures of taste and/or smell and measures of food behavior.

Study	Subjects and Cancer Type (*n*)	Study Design	Chemosensory Evaluation		Food Behavior Evaluation		Outcome
			Stimuli	Evaluation	Food Aspects	Method(s)	
Carson and Gormican, 1997 [20]	Breast (*n* = 14) or colon (*n* = 15); controls (*n* = 28)	Prospective, longitudinal cohort study; baseline and after 2 weeks of treatment	Sucrose, sodium chloride, hydrochloric acid, urea	Taste: DT and RT presented as oral drops	Appetite, amount eaten, consumption of specific menu items	Questionnaire (rated as increased, decreased, or no change)	Prior to treatment, there was a significant correlation between increased sucrose RT (decreased sensitivity) with reduced appetite within cancer patient groups
Trant et al., 1982 [21]	Upper gastrointestinal or lung (*n* = 62)	Cohort study post-treatment	Cherry drink with sucrose, tomato juice with sodium chloride, lemonade with citric acid, tonic water with urea (five conc. of each)	Taste: Intensity and hedonic rating on a 10-cm labeled analog scale (0–10)	Energy intake and number of servings	24-h dietary recall	Higher ratings for salt intensity associated with increased energy intake
Ovessen et al., 1991 [22]	Breast (*n* = 4), lung (*n* = 16), ovarian (*n* = 11)	Prospective, longitudinal; visits at baseline and after three treatment cycles (2–3 months postbaseline)	Anodal current (2.5–370 µA) using a gustometer	Taste: DT	Dietary intake	3-day food record	No relationship between taste or smell DT and food intake
			Pyridine	Smell: DT using squeeze bottles			
Pattison et al., 1997 [23]	Non-specified cancer type (*n* = 22); controls (*n* = 16)	Cross-sectional cohort study; visit not specified	Sweet, sour, salty, and bitter (compounds not stated)	Taste: DT (presentation method not stated)	Dietary intake	Weighted measurement of food intake for one meal on three different days	No relationship between taste DT measures and macronutrient intake
Sánchez-Lara et al., 2010 [24]	Unspecified cancer type (*n* = 30); controls (*n* = 30)	Cross-sectional cohort study; visit after second treatment cycle	Sucrose, urea, sodium glutamate	Taste: DT and RT using whole-mouth stimuli	Dietary intake	SNUT food frequency questionnaire	Patients with higher sucrose DT consumed less protein, carbohydrate, zinc, and overall calories; patients with higher urea RT consumed less protein, carbohydrate, fat, and overall calories
Boltong et al., 2014 [25]	Breast (*n* = 52)	Prospective longitudinal; visits: baseline, beginning, middle, and late in third cycle, beginning of last cycle, and 2 months after treatment	Sucrose, sodium chloride, citric acid, caffeine, and monosodium glutamate	Taste: Identification *: Method of investigating sensitivity of taste using whole-mouth stimuli	Dietary intake	Food frequency questionnaire	Deterioration in identification of all five taste qualities correlated with reduced energy intake
IJpma et al., 2016 [18]	Testicular (*n* = 50); controls (*n* = 48)	Cross-sectional cohort; visits 1, 3, 5, or 7 years after treatment	Sucrose, citric acid, quinine HCl, sodium chloride	Taste: RT using taste strips	Dietary intake food preference	Food frequency questionnaire; preference for pictures of snack products	No relationship between taste/smell scores and food preference or dietary intake
			*n*-butanol and a set of 16 common odors	Smell: DT, discrimination, identification, using Sniffin’ Sticks			
Turcott et al., 2016 [26]	Lung (*n* = 40)	Prospective longitudinal; visits: baseline, after two cycles	Sucrose, urea, sodium glutamate presented as whole-mouth stimuli	Taste: DT and RT	Dietary intake, appetite	SNUT food frequency questionnaire	Increase in sweetness sensitivity (i.e., lower DT from baseline) associated with decreased intake of protein, fat, and iron
IJpma et al., 2017 [27]	Testicular (*n* = 21); controls (*n* = 48)	Cross-sectional and prospective longitudinal; visits: baseline, before and after cycles 1 and 2, end of treatment, 7 and 12 months from start of treatment	Sucrose, citric acid, quinine HCl, sodium chloride	Taste: RT using taste strips	Dietary intake food preference	Food frequency questionnaire; preference for pictures of snack products	No relationship between taste/smell scores with food preferences or dietary intake
			*n*-butanol and a set of 16 common odors	Smell: DT, discrimination, identification, using Sniffin’ Sticks			
de Vries et al., 2017 [28]	Oesophago-gastric (*n* = 15)	Prospective longitudinal; visits at baseline and before start of third cycle	*n*-butanol and a set of 16 common odors	Smell: DT, discrimination, identification using Sniffin’ Sticks	Food preferences	MFPRT rating	No relationship between taste/smell and food preferences
			Sucrose, citric acid, sodium chloride, quinine HCl	Taste: RT using taste strips			
de Vries et al., 2018 [17]	Breast (*n* = 28); controls (*n* = 28)	Prospective longitudinal; visits at baseline, during treatment, and 1–3 weeks and 6 months after treatment	Sucrose, sodium chloride, citric acid, quinine HCl	Taste: RT using taste strips	Food preferences	MFPRT rating	No relationship between taste/smell and food preferences; no significant changes in ranking of sampled spiked beverages
			Kool-Aid with sucrose, tomato juice with sodium chloride (five conc. of each)	Taste: Preference (rank samples in order of liking)			
			*n*-butanol and a set of 16 common odors	Smell: DT, discrimination, identification using Sniffin’ sticks			

* ISO 3972:2011: International Organization for Standardization—method of investigating sensitivity of taste. DT—taste or odor detection threshold; MFPRT—macronutrient and food preference rating task (rating of food pictures); RT—taste or odor recognition threshold; SNUT—nutritional assessment system habits and nutrient intake (Spanish); conc.—concentration.

**Table 2:** Methods used to evaluate taste and smell function.

Method	Task	Stimuli	Studies
Taste			
Recognition threshold (RT)	Lowest concentration a taste can be recognized	Taste strips, whole-mouth solutions, or oral drops	[17,18,20,24,26,27,28]
Detection threshold (DT)	Lowest concentration something different from water is detected	Taste strips, whole-mouth solutions, or oral drops	[20,23,24,26]
Identification	Identify tastes from a single suprathreshold concentration	Whole-mouth solution	[25]
Intensity	Rating on a 10-cm analog scale labeled from “no (taste sensation)” to “extremely (taste sensation)”.	Beverages spiked with tastant	[21]
Liking	Rating on a 10-cm analog scale labeled from “dislike extremely” to “like extremely”	Beverages spiked with tastant	[21]
Electrogustometry	Lowest detected electrical stimuli (µA) (DT)	Anodal current via gustometer	[22]
Preference	Rank samples in order of liking	Beverages spiked with tastants at five concentrations	[17]
Smell			
DT	Lowest concentration detected	Pyridine or *n*-butanol (squeeze bottles or Sniffin’ Sticks)	[17,18,22,28]
Discrimination	From three odors, select the odor that is different from two other identical odors (16 triplets presented)	Sniffin’ Sticks	[17,18,28]
Identification	Identify the odor from a choice of four options (16 common odors)	Sniffin’ Sticks	[17,18,28]

**Table 3:** Effect sizes for taste – difference in food intake in cancer patients.

Taste	Food-Related	Intake ^a^ or Change in Intake ^b^ in Patients with Low DT (Mean ± SD).	Intake ^a^ or Change in Intake ^b^ in Patients with High DT (Mean ± SD).	Effect *d* (95% CI)	Reference
Sweet	Zinc (mg/day)	17 ± 7 ^a^	9.6 ± 4.5 ^a^	1.18 (0.41, 1.96)	Sánchez-Lara, 2010 [24]
Sweet	Fat (g/day)	+27.39 ^b^	−15.1 ^b^	0.97 (0.31, 1.62)	Turcott, 2016 [26]
Sweet	Carbohydrate (g/day)	240 ± 84 ^a^	167 ± 81 ^a^	0.88 (0.13, 1.63)	Sánchez-Lara, 2010 [24]
Sweet	Animal protein (g/day)	+18.6 ^b^	−15.1 ^b^	0.86 (0.21, 1.51)	Turcott, 2016 [26]
Sweet	Protein (g/day)	+30.8 ^b^	−7.9 ^b^	0.81 (0.16, 1.45)	Turcott, 2016 [26]
Sweet	Calories (kcal/day)	1970 ± 658 ^a^	1450 ± 833 ^a^	0.69 (−0.04, 1.43)	Sánchez-Lara, 2010 [24]
Sweet	Iron (mg/day)	+8.4 ^b^	−1.11 ^b^	0.65 (0.01, 1.29)	Turcott, 2016 [26]
Sweet	Appetite *			0.59 (0.00, 1.18)	Carson, 1997 [20]
Sweet	Protein (g/day)	74 ± 45 ^a^	53 ± 32 ^a^	0.54 (−0.19, 1.27)	Sánchez-Lara, 2010 [24]
Bitter	Calories/day	2124 ± 812 ^a^	1493 ± 452 ^a^	0.96 (0.20, 1.72)	Sánchez-Lara, 2010 [24]
Bitter	Carbohydrate (g/day)	254 ± 98 ^a^	182 ± 58 ^a^	0.89 (0.14, 1.64)	Sánchez-Lara, 2010 [24]
Bitter	Fat (g/day)	87 ± 38 ^a^	62 ± 22 ^a^	0.81 (0.06, 1.55)	Sánchez-Lara, 2010 [24]
Bitter	Protein (g/day)	83 ± 53 ^a^	52 ± 17 ^a^	0.79 (0.04, 1.53)	Sánchez-Lara, 2010 [24]

Effect size *d* represents the standardized difference of means in food intake, calculated based on mean, standard deviation (SD), *p*-value, and sample size provided in the literature, between patients with a low/decreased DT and patients with high/increased DT. CI—95% confidence interval. ^a^ Mean intake in patients with low DT. ^b^ Change in the mean intake in patients with a decreased DT. * The study only reported the number of patients with increased or decreased appetite after chemotherapy and the significance (*p*-value) of the difference. “+” denotes an increase in intake.

## Abbreviations

DT	Detection threshold
MFPRT	Macronutrient and food preference rating task
RT	Recognition threshold
SNUT	Nutritional assessment system habits and nutrient intake (Spanish)
